# The Influence of Inflammation on Anemia in CKD Patients

**DOI:** 10.3390/ijms21030725

**Published:** 2020-01-22

**Authors:** Anna Gluba-Brzózka, Beata Franczyk, Robert Olszewski, Jacek Rysz

**Affiliations:** 1 Department of Nephrology, Hypertension and Family Medicine, Medical University of Lodz, 90-549 Lodz, Poland; bfranczyk-skora@wp.pl (B.F.);; 2 Department of Geriatrics, National Institute of Geriatrics Rheumatology and Rehabilitation and Department of Ultrasound, Institute of Fundamental Technological Research, Polish Academy of Sciences, Warsaw, Poland (IPPT PAN), 02-106 Warsaw, Poland; robert.olszewski@me.com

**Keywords:** inflammation, chronic kidney disease, anemia, anemia of inflammation, ESA hyporesponsiveness

## Abstract

Anemia is frequently observed in the course of chronic kidney disease (CKD) and it is associated with diminishing the quality of a patient’s life. It also enhances morbidity and mortality and hastens the CKD progression rate. Patients with CKD frequently suffer from a chronic inflammatory state which is related to a vast range of underlying factors. The results of studies have demonstrated that persistent inflammation may contribute to the variability in Hb levels and hyporesponsiveness to erythropoietin stimulating agents (ESA), which are frequently observed in CKD patients. The understanding of the impact of inflammatory cytokines on erythropoietin production and hepcidin synthesis will enable one to unravel the net of interactions of multiple factors involved in the pathogenesis of the anemia of chronic disease. It seems that anti-cytokine and anti-oxidative treatment strategies may be the future of pharmacological interventions aiming at the treatment of inflammation-associated hyporesponsiveness to ESA. The discovery of new therapeutic approaches towards the treatment of anemia in CKD patients has become highly awaited. The treatment of anemia with erythropoietin (EPO) was associated with great benefits for some patients but not all.

## 1. Introduction

Anemia is frequently observed in the course of chronic kidney disease (CKD) and it is associated with a diminished quality of patients’ life. It also enhances morbidity and mortality and hastens the CKD progression rate [1]. Patients with CKD frequently suffer from a chronic inflammatory state which is related to the vast range of underlying factors, such as higher incidence of infections, increased levels of proinflammatory cytokines, the uremic milieu, the widespread presence of arteriosclerosis, and others [2]. The results of animal studies demonstrated that serum half-lives of proinflammatory cytokines, including TNF- and IL-1 are higher in animals without renal function [3]. Deterioration of renal function may also influence the level of other inflammatory molecules, such as serum C-reactive protein (CRP) or IL-6, which concentration inversely correlated with creatinine clearance [4,5].

The discovery of new therapeutic approaches towards the treatment of anemia in CKD patients has become highly awaited. Treatment of anemia with erythropoietin (EPO) was associated with great benefits for some patients but not all. Clinical and experimental evidence indicates the important role of inflammation in poor response to EPO therapy. Therefore, anti-inflammatory agents seem to be beneficial in the therapy of patients who are EPO bad responders as well as drugs that could antagonize the effects of hepcidin [6].

## 2. Anemia in CKD Patients

Anemia is a frequent problem in CKD patients [7]. In CKD, it is typically normocytic, normochromic, and hypoproliferative [8]. According to large-scale population studies the incidence of anemia (hemoglobin <12 g/dL) is less than 10% in patients with CKD stages I and II, 20%–40% in stage III, 50–60% in stage IV, and it exceeds 70% in patients with end-stage renal disease (stage V) [9,10,11]. Other studies indicate that in the dialysis population anemia incidence is as high as 90% [12,13].

The prevalence of more severe anemia (hemoglobin ≤ 10 g/dL) is much less common: 5.6% in stage 3 CKD and 27.2% in stage 5 non-dialysis CKD patients [13,14]. These data clearly show that anemia appears early in the course of CKD and its occurrence is increasing along with the declining glomerular filtration rate [11]. Data from the National Health and Nutrition Examination Survey (NHANES) in 2007–2008 and 2009–2010 [13] demonstrates that despite anemia being very common in patients with advanced CKD, patients in the United States were relatively rarely receiving treatment for it (only 20% of patients with CKD stage 4 and 42% of those with stage 5). Anemia in this study was defined using gender-specific thresholds (<12 g/dL for female patients and <13 g/dL for male patients). Glomerular filtration rate, gender, age, race, comorbidities are considered as the predictors of CKD anemia [15].

Anemia in patients with CKD is a multifactorial process, in which chronic inflammation, erythropoietin deficiency, iron metabolism disorders, blood loss on hemodialysis sessions, uncontrolled hyperparathyroidism, deficiency of essential nutrients like iron, folic acid, and vitamin B12, the use of some drugs, including ACE inhibitors and uremic toxins play the most important role [7,8,16,17]. The understanding of underlying mechanisms of anemia in CKD is important due to the fact that in some patients erythropoietin stimulating agents (ESA) treatment might be least ineffective or even deleterious [17,18].

*Pre-dialysis CKD patients.* According to studies, the relative deficiency in erythropoietin (EPO) production by the peritubular cells of kidneys is responsible for defective erythropoiesis in CKD patients [17,19]. Erythropoietin deficiency is associated with disturbances in the differentiation and maturation of red blood cell precursors [20]. The results of some studies imply that circulating uremic-induced inhibitors of erythropoiesis may contribute to the development of anemia [8,21]. Sera from uremic patients have been showed to inhibit hematopoietic progenitor’s growth [22]. Chiang et al. [23] demonstrated that indoxyl sulfate (IS), which is a protein-bound uremic toxin, impaired erythropoiesis in a hydroxylase inhibitor (HIF)-dependent manner and limited EPO gene transcription during hypoxia. It seems that IS stimulates hepcidin production via a pathway that involves both aryl hydrocarbon receptor (AhR) and oxidative stress, which in consequence leads to iron sequestration and impaired iron utilization in CKD [24]. Wu et al. [25] found that IS removal improved the impact of ESA on anemia in late-stage CKD patients confirming that IS mediates renal anemia via EPO regulation. Moreover, Ahmed et al. [26] suggested that indoxyl sulfate triggered suicidal erythrocyte death in renal failure. Radioisotope labeling studies confirmed shortened red blood cell survival due to metabolic and mechanical factors in CKD [21,27]. In CKD, it was shown that intra- and extracellular factors diminished red blood cell survival by 30% to 50%, probably due to the red blood cell membrane failure to pump sodium to the extracellular medium [28].

Apart from true iron deficiency, in many CKD patients, functional iron deficiency has been observed. It is characterized by disturbed iron release from body stores, which makes meeting the demand for erythropoiesis impossible. Low serum transferrin saturation (a parameter informing the amount of circulating iron) and normal or high serum ferritin (a marker of body iron stores) were shown in this group of patients. Secondary hyperparathyroidism, which is frequently observed in CKD, contributes to the development of anemia and greater resistance to erythropoietin [29]. Finally, hepcidin, which is a key regulator of circulating iron absorption, has been found to be involved in the etiology of anemia in CKD [30,31]. Its concentrations were demonstrated to be influenced by inflammation [32]. The fact that anemia develops in spite of elevated EPO levels in CKD suggests that peripheral resistance or hyporesponsiveness to EPO may be the factual reason for its occurrence [11].

*Dialysis CKD patients.* Apart from the aforementioned mechanisms, anemia in dialysis patients can be associated with iron losses. According to Besarab et al. [21], the losses of iron in hemodialysis patients reach 1–3 g per year and they are associated with chronic bleeding related to platelet dysfunction and also frequent phlebotomy, hemolysis, and blood retained in the extracorporeal circulation during dialysis. Additionally, patients undergoing dialysis show impaired dietary iron absorption [33,34]. HD patients with high CRP levels (>8 mg/L) have been shown to have lower iron absorption than patients with lower CRP levels [35]. According to studies, inflammation (via cytokines and bacterial lipopolysaccharide, LPS) regulates hepcidin expression and production in response to liver iron levels, hypoxia and anemia, which results in functional iron deficiency or enhanced ferritin and diminished transferrin production, shunting iron to the reticuloendothelial storage pool instead of delivery to erythrocyte precursors [36,37,38]. Inflammation acting together with uremic toxicity and hepcidin exacerbates anemia at different stages. Tozoni et al. [39] suggested that in HD patients, hypoxemia and uremic toxins may act synergistically and decrease red blood cell life span (RBCLS). They provided evidence that even in the case of healthy red blood cells, hypoxia, and uremia stimulated eryptosis and disturbances in redox balance. These two stimuli together have been demonstrated to increase PS exposure, stimulate cellular shrinkage, and enhance calcium influx into the RBC [39]. Bonomini et al. [40] revealed that the RBC life span in CKD patients was related to enhanced erythrocyte deformability and abnormalities of plasma membrane symmetry and cytoskeleton. These alterations and the destruction of the membrane were shown to be hastened by the uremic environment, inflammation, and oxidative stress [41]. However, Bataille et al. [17] failed to find any correlation between plasmatic concentrations of uremic toxins, such as indole 3-acetic acid (IAA), sulfate (IS), and paracresyl sulfate (PCS), and any parameter related to anemia in hemodialysis patients. Therefore, they suggested that indolic uremic toxins and PCS may have no or a very slight impact on anemia parameters, i.e., Hb concentrations or ESA hyporesponsiveness in this population [17].

### KDIGO Recommendations

KDIGO guidelines recommend hemoglobin level measurement in patients with CKD without diagnosed anemia when clinically indicated but at least once a year in subjects with CKD stage 3, at least 2 times a year in those with CKD stage 4–5 who are not on dialysis and at least every 3 months in those with stage 5 CKD undergoing either hemodialysis (HD) or peritoneal dialysis (PD) (Not Graded) [42]. In case of CKD patients with anemia, who are not treated with ESA, Hb levels should be measured when clinically indicated (Not Graded)—at least every 3 months in patients with CKD stage 3–5 who are not on dialysis (CKD-ND) or stage 5D on PD and every month in patients with CKD 5D in HD. In turn, in patients with anemia on ESA treatment, Hb levels should be measured when clinically indicated—in the correction phase once in a month and in the maintenance phase: at least every 3 months in patients with CKD (not on dialysis), monthly in patients with CKD-5D (hemodialysis), and every 2 months in patients with CKD patients on peritoneal dialysis [42].

The administration of vitamin D analogs has been linked with the amelioration of anemia and/or the reduction in EPO needs [29,43]. The beneficial effect of vitamin D may be associated with its impact on the suppressive effect of PTH and/or the stimulation of erythrocyte progenitor cells [29,43].

Clinical observations indicate that anemia in patients with end-stage renal disease (ESRD) and non–dialyzed CKD subjects is associated with poor outcomes, including higher mortality [14,44,45]. Large, retrospective studies carried by Yang et al. [46] and Brunelli et al. [47] have demonstrated the association between greater Hb variability and decreased survival. Another retrospective study of HD patients revealed a visible trend of increased mortality with increasing time below Hb target of 11 g/dL level [48]. In the case of patients whose Hb levels were <11 g/dL for 80%–100% of the time, mortality risk was ∼1.8 times as high as in patients with no time below this level. The Dialysis Outcomes and Practice Patterns Study (DOPPS) demonstrated that mortality and hospitalization risk declined by 5% and 6% per 1-g/dL higher patient baseline Hb level (*p* < or = 0.003 each), respectively. Moreover, risks of mortality and hospitalization risks were 10% to 12% lower for every 1-g/dL increased facility mean Hb level [49]. The results of these studies suggest that the maintenance of the target Hb range in CKD patients is an important goal in the treatment of renal anemia.

## 3. Inflammation in CKD and Anemia of Inflammation

The prevalence of inflammation in CKD patients varies between populations [50]. According to estimations, more than 30%–50% of patients with ESRD have serological evidence of an active inflammatory state, as indicated by elevated levels of CRP and pro-inflammatory cytokines, including IL-1, IL-6, and tumor necrosis factor (TNF-α) [51,52,53,54,55]. Among factors that are associated with the persistence of low-grade inflammation in CKD patients, there are oxidative stress, infectious complications, diminished clearance of cytokines, and dialysis-related factors [51]. The presence of more elevated levels of inflammatory markers, including serum ferritin and C-reactive protein was observed in patients with malnutrition in comparison to those without malnutrition (301.2 ± 127.1 mg/dL vs. 212.7 ± 124.9 mg/dL, *p* < 0.05; 63% vs. 33%, *p* < 0.05) [56]. Numerous studies indicated that the inflammatory state influences the development of renal anemia. de Francisco et al. [38] demonstrated that lower average CRP values were associated with better Hb control (*p* < 0.0001). Moreover, Agarwal et al. [57] revealed that serum albumin (an alternative inflammatory marker) was also a vital predictor of baseline Hb and sensitivity to ESAs. Proinflammatory cytokines have been shown to simultaneously affect erythropoiesis at several levels, including the suppression of erythroid progenitor cell proliferation [11]. Allen et al. [58] showed inhibition of erythroid colony formation by soluble factors in serum from patients with both end-stage renal disease and inflammatory disease. In vivo studies confirmed that the administration of TNF-α promotes hypoproliferative anemia through a direct effect on erythroid progenitor cells and indirect stimulation of IFN-γ production [59]. However, some other studies have provided contradictory results and suggested that TNF-α and IL-1 promoted the growth of early progenitors (burst-forming units) but they inhibited the growth at later stages of erythropoiesis, i.e., erythroid colony-forming units [60]. Inflammatory state affects erythropoiesis also via the inhibition of hypoxia-induced EPO production in Hep3B cells [61]. It seems that the key pathway through which inflammation promotes the development of anemia is the modulation of iron metabolism. Elevated ferritin levels, diminished iron, and iron-binding capacity, as well as higher abundance of iron in the bone marrow, are the characteristic features of inflammation-associated anemia [38]. These features imply iron sequestration in reticuloendothelial cells and the state of inadequate plasma iron levels to support erythropoiesis [62]. The exact mechanism of the influence of the inflammatory state on the development of anemia may be related to hepcidin which affects iron homeostasis via the binding of the cell surface iron transporter ferroportin [63]. Consequently, the phosphorylation, internalization, ubiquitination, and degradation of ferroportin in the lysosomes is triggered [11,62]. This results in decreased iron efflux from duodenal enterocytes into the circulation (reduced iron absorption) as well as the diminished release of iron from macrophages into the reticuloendothelial system and finally in hypoferremia. The results of animal studies have indicated that in transgenic mouse models hepcidin is a key negative regulator of iron absorption in the small intestine, and iron release from macrophages [64]. Wrighting and Andrews [65] demonstrated that interleukin-6 induced hepcidin expression through signal transducer and activator of transcription 3 (STAT3). It has been shown that during inflammation increased hepcidin levels limited iron release from enterocytes, hepatocytes, and macrophages thus decreasing its availability for bacteria [2,64]. Dallalio et al. [66] indicated that the influence of hepcidin on the development of anemia of inflammation involved not only the impact on iron metabolism but also the inhibition of erythroid progenitor proliferation and survival. Numerous studies confirmed the causal role of hepcidin in the process of anemia of inflammation [11,67,68,69]. Sasu BJ indicated [68] that neutralizing monoclonal antibodies to hepcidin along with ESA restored normal hemoglobin levels in a mouse model of bacteria-induced anemia of inflammation, while ESA administration alone was not effective. The administration of exogenous EPO has been suggested to reduce hepcidin levels and therefore to ameliorate anemia of inflammation and iron sequestration [70]. Marked suppression was observed after 24 h from the administration of EPO and it persisted for a week.

Higher levels of inflammatory markers have been found to be related to decreased survival of CKD patients. Kalantar-Zadeh et al. [71] demonstrated 1.14 [95% confidence interval (CI) 1.03–1.26, *p* = 0.01] adjusted hazard ratio for death for each 1000 pmol/L increase in serum levels of myeloperoxidase (MPO) in 356 patients on maintenance HD. In the Modification of Diet in Renal Disease (MDRD) study involving stage 3 and 4 CKD patients, high CRP (≥0.3 mg/dL) was an independent predictor of both all-cause mortality and cardiovascular mortality in comparison to low CRP <0.3 mg/dL groups [72]. Other studies confirmed the relation between significantly increased overall mortality and cardiovascular mortality of HD patients and elevated CRP levels when compared to normal CRP levels (*p*  <  0.0001) [73,74]. Increased concentrations of IL-6 in incident dialysis patients were also found to be considerably associated with poor outcome [53].

## 4. Anemia Treatment

### 4.1. Anemia Treatment with Iron

Anemia treatment in CKD patients should be based on drugs that enhance the synthesis of erythrocytes and provide adequate levels of iron for hemoglobin formation [75,76]. According to National Institute for Health and Care Excellence (NICE) the treatment of anemia in CKD patients requires the use of either iron or erythropoiesis-stimulating agents, their combination in order to address both absolute and functional iron deficiency [77,78]. According to the KDIGO guideline 2012, the correction of iron deficiency with oral or intravenous iron supplementation can reduce the severity of anemia in patients with CKD [42]. Physician prescribing iron therapy should balance the potential benefits of avoiding or minimizing blood transfusions, ESA therapy, and anemia-related symptoms against the risks of harm in individual patients, such as anaphylactoid and other acute reactions, unknown long-term risks) (Not Graded) [42]. Improving Global Outcomes (KDIGO) guidelines also suggest that in adult CKD patients with anemia who are not on iron or ESA therapy but also in adult CKD patients on ESA therapy who are not receiving iron supplementation, a trial of IV iron or alternatively 1–3 month trial of oral iron therapy (in non-dialysis CKD patients) (2C) should be introduced if patients require an increase in Hb concentration without starting ESA treatment or TSAT is ≤30% and ferritin is ≤500 ng/mL (≤500 mg/L) [42]. The route of iron administration should be selected on the basis of the severity of the iron deficiency, availability of venous access, response to prior oral iron therapy, side effects with prior oral or IV iron therapy, patient compliance, and cost. (Not Graded) [42]. The supplementation of oral iron is the simplest and cheapest iron deficiency therapy; however, it is frequently ineffective in CKD patients [79]. Oral preparations of iron (e.g., ferrous sulfate) are not appropriate in CKD patients due to impaired intestinal iron absorption and side-effect in the form of abdominal discomfort, constipation, and nausea [42]. In turn, IV iron improves medication adherence and the efficacy of iron deficiency treatment but requires IV access and is associated with infrequent but severe adverse reactions [42].

In patients with CKD ND, IV iron administration is preferred due to the fact the available evidence supports its better efficacy in comparison to oral administration of iron; however, due to the fact that the difference in the effect is rather small, in these patients, the route of iron administration can be either IV or oral [42,80,81,82]. Oral iron is typically prescribed to provide approximately 200 mg of elemental iron daily. However, in some patients, smaller daily doses may be useful and better tolerated. If the goals of iron supplementation are not met with a 1–3-month course of oral iron, IV iron supplementation should be considered [42]. The evidence derived from RCTs and other studies comparing IV iron with oral iron and placebo supports IV iron administration in CKD 5HD patients as it is associated with a greater increase in Hb concentration, a lower ESA dose, or both [42,83,84]. IV iron administration has been demonstrated to boost erythropoiesis, effectively replenish iron stores and enable the decrease of required ESA dose, however, it also promotes oxidative stress, atherosclerotic plaque development, and increases cardiovascular mortality [85]. Iron overload itself might be a cause of inflammation and contribute to ESA resistance. It has been showed to increase the synthesis of hepcidin which may be the link between inflammation and anemia [38,86].

### 4.2. Anemia Treatment with ESA

The exclusion of other than CKD causes of anemia, including iron and other hematinic deficiencies, chronic inflammation, malignancy, and drugs should be performed before the initiation of appropriate treatment [87]. Following the ruling out reversible causes of anemia, supplementary erythropoietin (epoetin) administration can be considered. Before the initiation and maintaining of ESA therapy, the potential benefits of reducing blood transfusions and anemia-related symptoms should be weighed against the risks of harm in individual patients (e.g., stroke, vascular access loss, hypertension) (1B) [88]. The decision concerning the initiation of ESA therapy in adult CKD ND patients with Hb concentration <10.0 g/dL (100 g/L) should be based on the rate of fall of Hb concentration, prior response to iron therapy, the risk of needing a transfusion, the risks related to ESA therapy, and the presence of symptoms attributable to anemia (2C) [88]. In case of adult CKD 5D patients, ESA therapy should be used to avoid Hb concentrations falling below 9.0 g/dL (90 g/L) by starting ESA therapy when the hemoglobin is between 9.0–10.0 g/dL (90–100 g/L) (2B) [88]. In the rest of CKD patients, ESA treatment is recommended in a dose enabling the maintenance of hemoglobin levels no higher than 11.5 g/dL (2B) [88]. According to recommendations, in all adult patients, ESAs should not be used to intentionally increase the Hb concentration above 13 g/dL (130 g/L) (1A), as it may increase the risk of stroke [18], hypertension [89], vascular access thrombosis (in case of hemodialysis patients) [90], and it can be associated with higher mortality [89]. The re-adjustment of ESA dose is required in patients suffering from ESA-related adverse events, in those with comorbidities resulting in ESA hyporesponsiveness or when Hb target range has been reached [88]. Numerous randomized clinical trials have demonstrated that Hg values ≥ 11.5 g/dL (≤115 g/L) in adult CKD patients may bring more harm than benefit [88]. Standard anemia treatment in CKD patients involves the administration of recombinant human erythropoietin, including epoetin α and epoetin β, due to the fact that a decrease in erythropoietin production in the kidneys is the key reason which underlines anemia [91]. Continuous erythropoiesis receptor activators which are a pegylated form of recombinant human erythropoietin with extended serum half-life allowing for longer dosing intervals (every 2 weeks) is currently gaining popularity in the community of dialysis patients [92]. Generally, the initial doses of epoetin-alfa or epoetin-beta dosing are from 20 to 50 IU/kg body weight three times a week. Darbepoetin-alfa doses usually start from 0.45 μg/kg body weight once weekly (subcutaneous or intravenous administration), or 0.75 μg/kg body weight once every 2 weeks (SC administration). In turn, CERA dosing starts at 0.6 μg/kg body weight once every 2 weeks by SC (CKD ND) or IV administration (CKD 5D patients), or 1.2 μg/kg body weight once every 4 weeks by SC administration for CKD ND patients [88]. Moreover, when a downward adjustment of Hb concentration is needed the decreasing of ESA dose instead of its withholding is suggested (2C). The frequency of ESA administration should be based on the CKD stage, patient tolerance, treatment setting, efficacy, and type of ESA. According to recommendations, during the initiation phase of ESA therapy, Hb concentration should be measured at least monthly. Later, during the maintenance phase, Hb level in non-dialysis CKD patients should be measured at least every 3 months, while in HD patients at least monthly. Treatment with ESA has been demonstrated to alleviate fatigue, weakness and headaches, improve quality of life and neurocognitive function as well as to lower the frequency of necessary blood transfusions [88]. A randomized, controlled trial performed by the Canadian Erythropoietin Study Group which included HD patients randomized to three groups to receive placebo (*n* = 40), erythropoietin to achieve a hemoglobin concentration of 95–110 g/L (*n* = 40), or erythropoietin to achieve a hemoglobin concentration of 115–130 g/L (*n* = 38) demonstrated significant improvements in fatigue, physical function, moderate improvements in exercise tolerance and depression in ESA treated patients in comparison to patients not receiving erythropoietin. No differences were found in the abovementioned parameters between high and low hemoglobin groups [93]. However, three large randomized controlled trials (The Normal Hematocrit Study (NHCT) [90], The Correction of Hemoglobin and Outcomes in Renal Insufficiency (CHOIR) trial [94], and The Trial to Reduce Cardiovascular Events with Aranesp Therapy (TREAT) [18] demonstrated that establishing and reaching higher hemoglobin targets may be harmful to patients [92]. The re-analysis of results obtained in two large trials (CHOIR and the Cardiovascular Risk Reduction by Early Anemia Treatment (CREATE)) trials revealed that patients who despite receiving higher doses did not achieve their target hemoglobin had worse outcomes [95,96]. The use of too high doses of EPO was associated with an increased risk of cardiovascular incidents, stroke, rapid malignant progression in cancer patients, pure red blood cell aplasia, and increased mortality in other patients [90,97]. The abovementioned results of randomized controlled trials resulted in establishing KDIGO guidelines, which suggest that ESA should be administered with great caution in the case of CKD patients with active malignancy (1B), a history of stroke (1B), or a history of malignancy (2C) [88]. Patients treated with epoetin frequently require supplementation with oral or intravenous iron to maintain sufficient iron stores during the correction and the maintenance phases of management [14]. ESA treatment enhances erythropoiesis which leads to the exhaustion of iron pool, resulting in a relative iron deficiency [8]. The inflammatory state observed in CKD hinders erythropoiesis and reduces iron availability by the production of hepcidin [98,99]. Due to the fact that CKD patients also suffer from greater blood loss and diminished intestinal absorption of dietary iron, the supplementation of this compound is important to prevent absolute iron deficiency [76].

### 4.3. New Strategies of Anemia Treatment

New therapies targeted at inhibiting hepcidin production are being investigated as potential anemia treatment [76]. Studies are performed to assess the efficacy and safety of anti-IL-6 antibodies such as Tocilizumab and IL-6 monoclonal antibodies such as sultuximab. The latter one has been shown to increase hemoglobin levels, however, at the same time, it enhanced the risk of infections [100]. The administration of Atorvastatin to CKD was shown to significantly lower serum hepcidin levels and improved hematological parameters [101]. It has been demonstrated that activin type-II receptor (ActRII) IgG-Fc fusion proteins, including sotatercept and luspatercept, increase red blood cell numbers and hemoglobin levels in humans [102]. Activins are soluble ligands belonging to a large transforming growth factor-β (TGF-β) family and their expression is observed in bone marrow cells, including erythroid cells [103]. They are involved in the proliferation and differentiation of embryonic/hematopoietic stem and erythropoietic cells. Sotatercept is a fusion protein comprising of an extracellular chain of activin receptor IIA and the Fc domain of human IgG1. It inhibits the activation of endogenic, membranous receptors (ActRIIA) of activin by binding circulating activin and related proteins (e.g., BMP 10 and BMP 11) [104] Moreover, it influences the expression of angiotensin II which can promote erythropoiesis directly and indirectly via EPO production [105]. Sotatercept also stimulates the release of the mature erythrocyte forms, decreases the expression of the vascular endothelial growth factor (VEGF), which is an inhibitor of erythropoiesis and inhibits hepcidin transcription in the liver [106]. The results of preliminary studies with sotatercept in dialysis patients have demonstrated a dose-dependent increase in Hb and a decrease in extraosseous calcification [107]. Luspatercept ACE-536; Acceleron/Celgene Corp) is another ligand-trapping fusion protein that contains the extracellular domain of human activin receptor type IIB (ACTRIIB) modified to diminish activin binding [108]. In vivo, it has been shown to exert erythropoietic activity and stimulate the maturation of late-stage erythroid precursors in vivo [109]. The treatment with EPO and luspatercept provides a synergistic erythropoietic response [109]. In a phase 1, randomized, double-blind, placebo-controlled, clinical trial of ACE-536, it increased Hb levels in a dose-dependent mode 7 days after treatment initiation, and this effect was maintained for several weeks following treatment in postmenopausal women [110]. These observations are supported by an ongoing phase 2 clinical trials of ACE-536 in patients with β-thalassemia and myelodysplastic syndromes [108]. In clinical trials, these novel compounds were found to be well tolerated by healthy volunteers and patients suffering from anemia due to CKD, however, they have not been approved for sale as therapeutics as their long-term efficacy and safety especially the issues of immunogenicity and antifibrotic effects, still needs to be confirmed. Sasu et al. [111] using human hepcidin (hHepc) knock-in mice as a model of inflammation-induced anemia showed that high-affinity antibodies specific for hHepc neutralized hHepc in vitro and in vivo and facilitated anemia treatment due to the fact that they enhanced the absorption of dietary iron and stimulated its mobilization from iron stores for use in erythropoiesis [112]. In the treatment of anemia, CKD also compounds targeting hypoxia-inducible factor prolyl hydroxylase inhibitor (Hif1α inhibitors), including Vadustat, Daprodustat, and Roxadustat, have been studied. HIF1α seems to be an interesting target as it regulates renal EPO production and erythropoiesis [76]. The results of phase 2 trials involving CKD patients indicate that roxadustat enhanced levels of endogenous erythropoietin to within or near the physiologic range, and also it increased hemoglobin levels and improved iron homeostasis [113,114,115,116,117]. A single-blind, placebo-controlled study of ND-CKD stage 3 or 4 patients randomized to receive four escalating doses (0.7, 1.0, 1.5, 2.0 mg/kg) of roxadustat either twice or thrice weekly over 28 days demonstrated that this drug increased Hb in a dose-dependent manner [114]. Oral administration of 1 mg/kg roxadustat twice-weekly resulted in the increase in endogenous EPO (eEPO) levels after 4 h, with its peak at ~10 h, and the return to baseline within 24–48 h. In a subsequent phase IIa open-label study, analyzing various roxadustat dose regimens for 16 and 24 weeks in NDD-CKD participants, 92% of patients achieved hemoglobin response [116]. The rise in the hemoglobin level was independent of baseline C-reactive protein levels and iron repletion status. 16-week treatment with roxadustat resulted in the reduction in hepcidin levels by 16.9% (*p* = 0.004) and the increase in hemoglobin level by a mean (±SD) of 1.83 (±0.09) g/dL (*p* < 0.001), while reticulocyte Hb content remained the same [116]. TSAT and ferritin levels diminishing was observed during the initial weeks of treatment with roxadustat, however, later they were stabilized [116]. These results were similar to those obtained in an open-label, phase IIb study, ESA-naïve incident PD and HD participants with severe anemia (mean Hb 8.3 g/dL at baseline) who were randomized to receive no iron, oral iron, or IV iron during the treatment with roxadustat for 12 weeks [115]. In this study, in 96% of patients, the Hb response (increase in Hb of ≥1.0 g/dL from baseline) was observed. Roxadustat treatment resulted in Hb elevation of ≥2 g/dL within 7 weeks of treatment, which was independent of baseline Hb level, iron repletion status, inflammatory status, and dialysis modality. A greater Hb response was found in groups of patients receiving also iron in comparison to those not receiving iron. Mean serum hepcidin was decreased significantly after 4 weeks of study [115]. Third phase trial in which CKD patients with Hg levels of 7.0 to 10.0 g/dL were randomly assigned to receive roxadustat or placebo three times a week for 8 weeks an increase of 1.9 ± 1.2 g/dL in the roxadustat group and a decrease of 0.4 ± 0.8 g/dL in the placebo group (*p* < 0.001) was observed. Moreover, a reduction from baseline in the hepcidin level by 56.14 ± 63.40 ng/mL in the roxadustat group and 15.10 ± 48.06 ng/mL in the placebo group was seen [113]. However, in the group receiving roxadustat, hyperkalemia, and metabolic acidosis were more frequent than in the placebo group. The beneficial impact of roxadustat on hemoglobin level maintained during the 18-week open-label period. According to the authors, the stability of serum iron levels in the roxadustat group may have been related to reductions in hepcidin levels, which enabled gut absorption of iron and improved the release of macrophage iron onto transferrin [113,118]. Other studies of non-dialyzed CKD patients have demonstrated that roxadustat increased hemoglobin levels with stable serum iron levels, despite robust erythropoiesis in the absence of intravenous administration of iron [116,119]. Moreover, it has been revealed that roxadustat is superior to the placebo in correcting anemia in non-dialysis CKD patients, it is non-inferior to erythropoietin-α for treatment of anemia in long-term dialysis patients.

Molidustat is another potential alternative to the standard treatment of anemia associated with CKD as it increases erythropoietin production and improves iron availability. This HIF-PH inhibitor mimics hypoxia by stabilizing HIF-α subunits and it shows high relative selectivity for the induction of EPO gene expression, predominately in the kidney [120,121]. Molidustat enables the accumulation of HIF, which is then transported to the nucleus where it promotes the transcription of EPO and other hypoxia-inducible genes and thus leading to the elevation of endogenous EPO levels [122].

In preclinical studies, molidustat restored renal EPO production with minor stimulation of hepatic EPO [121,123]. Moreover, it heightened plasma EPO and EPO mRNA in the kidney prevented the reduction in hematocrit and corrected Hb level [121]. A single-center, randomized, single-blind, placebo-controlled, group-comparison, dose-escalation 1 phase study demonstrated that oral administration of molidustat to healthy volunteers elicited a dose-dependent increase in endogenous EPO and that all doses of molidustat were well tolerated [124]. In three randomized, controlled, phase 2 studies, which are the part of the DIALOGUE (DaIly orAL treatment increasing endOGenoUs Erythropoietin) program, molidustat diminished transferrin saturation (TSAT), hepcidin, ferritin, and iron concentrations and increased total iron-binding capacity (TIBC) in treatment-naïve patients not on dialysis [125]. In these studies, the efficacy, safety, and tolerability of molidustat were compared with placebo or alternative ESA therapy in patients with anemia of CKD. In the first fixed-dose, placebo-controlled study (DIALOGUE 1), molidustat was shown to increase hemoglobin levels in patients not on dialysis [121]. The efficacy of molidustat was confirmed in DIALOGUE 2, in which patients were switched from darbepoetin to molidustat or continued with darbepoetin. Molidustat in all dose arms enabled maintaining hemoglobin levels within the pre-specified target range of 10.0–12.0 g/dL. The results indicate that starting dose of 25 or 50 mg once daily seems to be appropriate for CKD patients, since higher doses (i.e., 75 mg once daily) may increase the probability that hemoglobin levels will rise above the pre-specified limits [121]. In turn, in dialysis patients (DIALOGUE 4), only starting doses of molidustat of 75 and 150 mg once daily effectively maintained hemoglobin levels within the target range after switching from epoetin. Despite the level of kidney function impairment and disturbed hepatic erythropoietin production in included patients, molidustat mainly addresses kidney erythropoietin production [121]. In this study, patients treated with molidustat starting doses of 75 or 150 mg once daily had lower response rates, spent less time within the target hemoglobin range, and were more likely to have hemoglobin levels above the pre-specified limit in comparison to epoetin group [120,121]. Therefore, it seems that molidustat is an effective alternative to rhEPO and its analogs in the long-term management of anemia associated with CKD [122].

However, the treatment with HIF-PH inhibitors raises some safety concerns, due to the fact that these agents may stimulate tumorigenesis and angiogenesis which may exert a negative effect on retinal diseases or cancer [126]. Moreover, in phase 2 studies of these drugs, cases of hyperkalemia, hyperglycemia, and hyperuricemia were reported. It seems that adverse events in CKD patients may be related to the pharmacokinetics and dosing of HIF-PHIs. Finally, it has been suggested that these drugs may promote the development of thromboembolic complications such as pulmonary hypertension as well as the progression of CKD and polycystic kidney disease [126].

Pentoxifylline (methylxanthine derivative, PTX) is another drug which efficacy in the treatment has been tested in the CKD population [79]. Cooper et al. [127] hypothesized that pentoxifylline, which is traditionally used in the treatment of peripheral vascular disease, might improve the response to ESAs in anemic CKD patients via the inhibition pro-inflammatory cytokine production and thus the enhanced erythropoiesis. In their study, the use of oral pentoxifylline for 4 months in patients with ESRD and ESA-resistant anemia considerably increased the Hb concentration (*p* = 0.0001). Benbernou et al. [128] studied the effect of pentoxifylline on T-helper cell-derived cytokine production in human blood cells. Their study indicated that at an appropriate concentration (5 × 10^−4^ M concentration), PTX selectively suppressed interleukin-2 (IL-2), and interferon-gamma (INF-γ), while high levels of this drug (1 × 10^−3^ M) inhibited both TH1- and TH2-derived cytokines.

The results of phase 2 placebo-controlled studies in patients with CKD treated with vadadustat [129] as well as placebo-controlled [130] and dose-ranging [131] study of daprodustat in CKD population have demonstrated that such treatment was associated with the increase in hemoglobin level and its maintaining.

Vadadustat (AKB-6548) has been shown to restore baseline eEPO levels within 24 h following its oral administration [132]. In a phase IIa, double-blind, placebo-controlled trial of CKD patients who were randomized to receive escalating doses (240, 370, 500, 630 mg) of vadadustat or placebo orally once daily for 6 weeks, vadadustat significantly enhanced Hb levels in a dose-dependent manner. Moreover, it increased the total iron-binding capacity and decreased concentrations of ferritin and hepcidin. A phase IIb double-blind, placebo-controlled trial of non-dialyzed CKD patients randomized to receive a titratable dose of vadadustat (initial dose 450 mg) or placebo once daily for 20 weeks assessed efficacy and safety of once-daily vadadustat [129]. In this study, 54.9% of patients on vadadustat and 10.3% of patients on placebo achieved or maintained either a mean hemoglobin level of 11.0 g/dL or more or a mean increase in hemoglobin of 1.2 g/dL or more over the pre-dose average. Moreover, reticulocytes and total iron-binding capacity increased considerably in patients receiving vadadustat, while serum hepcidin and ferritin levels were reduced in comparison to patients on placebo. The authors concluded that vadadustat raised and maintained hemoglobin levels in a predictable and controlled manner while enhancing iron mobilization in patients with nondialysis-dependent CKD [129].

The effectiveness of daprodustat in the treatment of CKD-related anemia has been evaluated. In a 28-day, double-blind, phase IIa study of CKD stage 3–5 patients (*n*  =  73) randomized to receive fixed daprodustat doses 0.5 mg, 2 mg, and 5 mg once daily or placebo [130], the treatment resulted in a dose-dependent increase in Hb and also dose-dependent decrease in hepcidin concentrations. In a second, parallel phase IIa conversion study comprising 83 HD participants maintained on stable doses of rhEPO who were randomized to receive the same doses of daprodustat as the prior study or to continue rhEPO [130], only the administration of 5 mg of daprodustat allowed maintaining Hb levels similarly to rhEPO; however, in groups receiving lower doses a reduction of Hb levels at 4 weeks was observed. Moreover, hepcidin levels increase was demonstrated in daprodustat low doses groups. Its concentration remained the same in patients receiving 5 mg of the drug and decreased in the rhEPO arm at 4 weeks.

New drugs in the treatment of anemia have been summarized in Table 1.

According to studies, also vitamin D has also decreased hepcidin gene transcription, reduced serum levels by 50% in healthy individuals within 24 h, stimulated erythropoiesis and limited inflammation [133]. Zughaier et al. [134] confirmed that in early-stage CKD patients, vitamin D3 supplementation lowered the hepcidin level after three months of the administration. However, this effect was not observed when the calcitriol form of vitamin D was used in patients with mild to moderate CKD [135]. Therefore, further studies are needed to confirm the effects of vitamin D in CKD patients. The use of vitamin E-modified dialysis membranes in ESA-treated HD patients was also shown to improve anemia. This phenomenon was associated with concentration-dependent vitamin E-related improvement of red blood cell survival [136].

Apart from new drugs, the improvement of anemia may be achieved in HD patients by greater adequacy of hemodialysis measured by Kt/V (which mirrors the clearance of urea and it is a surrogate marker for the clearance of small, but not middle or large-sized, uremic toxins [79]). Equilibrated Kt/V A is a more accurate measure of the dialysis dose due to the fact that it corrects for urea rebound. Adequate dialysis has been shown to ameliorate anemia and decrease ESA dosage required for anemia correction in patients with ESRD [137,138,139]. Such an approach enables the correction of oxidative stress and the removal of molecules that inhibit erythropoiesis and erythrocyte G6PD activity [140]. Therefore, patients with adequate HD (Kt/V ≥ 1.2) have significantly higher erythrocyte G6PD activity and hemoglobin levels in comparison to patients who received inadequate HD [137]. Locatelli et al. [141] demonstrated that the use of a large-pore biocompatible membrane for a fixed 12-week follow-up improved anemia in hemodialysis patients in comparison with the use of a conventional cellulose membrane. Pedrini et al. [142] analyzed retrospectively the courses of hemoglobin levels and monthly ESA consumption in patients on mixed-HDF (hemodiafiltration) and on post-HDF. In Mixed-HDF, pre- and post-dilution substitution rates are adjusted by means of a feedback control system to obtain the maximal filtration fraction within safe pressure and hydraulic conditions, thus preventing progressive hemoconcentration [142,143,144]. Pedrini et al. [142] suggest that patients on mixed-HDF may have clinical benefits in terms of anemia management, including the requirement of lower ESA doses to maintain hemoglobin (Hb) levels within the recommended range. The use of Mixed-HDF enabled the maintaining of stable hemoglobin values with lower ESA doses when compared to Post-HDF patients. In their study, the monthly median ESA consumption of patients on Mixed-HDF at the end of the observation period was 50% lower than those of patients on Post-HDF. Authors suggested that this finding might be associated with the efficient removal of middle and large sized uremic toxins contributing to impaired erythropoiesis in dialysis patients. Maduell et al. [145] demonstrated a considerable amelioration of anemia when the substitution rate was substantially enhanced as a result of a better removal of uremic toxins. It seems that hepcidin is one of the important metabolites removed in such a dialysis. Stefansson et al. [146] confirmed that HDF removes hepcidin more efficiently than conventional HD, which results in clinical benefits related to anemia observed in HDF-treated patients. Moreover, the removal of proinflammatory cytokines also has been shown to be of high importance in the improvement of anemia as inflammatory cytokines can impair erythropoiesis and contribute to ESA resistance in CKD [142,147,148]. Some studies have demonstrated that HDF has the potential to reduce inflammation [149,150]. Other studies indicated lower ESA resistance index (ERI; (ESA/weight)/Hb [UI/kg/week/hb]) in patients treated with convective dialysis technique in comparison to patients treated with conventional HD [151,152]. However, some studies provided conflicting results in the context of anemia management and treatment modality is as other studies did not find improved anemia parameters in patients treated with convective dialysis technique [153,154]. Finally, a randomized clinical study designed to examine the effects of removal of inhibitors of erythropoiesis on anemia and EPO requirements in patients who could not reach target hemoglobin (Hb) levels (≥11 g/dL) despite treatment with subcutaneous EPO revealed significantly lower EPO doses in polysulphone high-flux dialyzer (HF-HD), and considerably increased Hb levels in comparison to polysulphone low-flux dialyzer (LF-HD) group [155]. In the HF-HD group, the reduction of beta2-microglobulin (b2-MG) and phosphorus levels during dialysis was significantly higher in comparison to the low-flux group (*p* < 0.001). The authors suggested that the beneficial effects of high-flux dialysis may be mediated by greater clearance of moderate and high molecular weight toxins.

## 5. The Impact of Inflammation on Response to Iron Supplementation and ESAs

As it has been mentioned above, anemia of inflammation is characterized by increased ferritin levels, diminished iron and iron-binding capacity (transferrin) and the presence of iron in bone marrow macrophages, which indicate disturbed mobilization of iron from stores [57]. According to studies inflammation diminishes predictive values of ferritin and hepcidin for iron status and responsiveness to iron therapy [156]. Inflammation-mediated elevation in hepcidin concentration results in iron trapping within the macrophages and hepatocytes, resulting in functional iron deficiency (FID) and the requirement of a higher dose of IV iron to maintain Hb targets [157,158]. On the other hand, too aggressive intravenous iron therapy (IIT) may boost inflammation in patients with end-stage renal disease (ESRD) and lead to subsequent disturbances of iron metabolism [159]. Moreover, it has been demonstrated that in HD patients with high CRP levels intestinal iron absorption is lower, probably as a result of an inflammation-induced increase in ferritin and hepcidin that block iron absorption [35,160].

Numerous studies have demonstrated that some CKD patients treated with ESAs respond poorly or not at all [161,162]. Hyporesponsiveness to erythropoiesis-stimulating agents occurs in approximately 5–10% of patients receiving ESA and it poses an important diagnostic and management challenge [163]. Among the most frequent causes of ESA resistance, there are non-compliance, absolute or functional iron deficiency, and inflammation. According to NKF-KDOQI guidelines, hyporesponsiveness to erythropoietin can be defined as the presence of at least one of the following three conditions: a major decrease in Hb level at a constant ESA dose, a considerable increase in the ESA dose requirement to maintain a given Hb level, or a failure to increase Hb level to greater than 11 g/dl despite an ESA equivalent to erythropoietin greater than 500 IU/kg/week [164]. The reason for poor responding to ESA may be associated with an enhanced inflammatory state with elevated levels of inflammatory markers, including C reactive protein (CRP), IL-1, IL-6, and TNF-α in CKD patients [165]. Cytokines may impair iron metabolism, which results in functional iron deficiency [38]. They also directly influence different erythropoiesis stages and mediate apoptosis induction, which implies that the cytokine-mediated pro-inflammatory signaling also affects EPO activity [166]. Cytokines inhibit the expression and regulation of specific transcription factors that are involved in the control of erythrocyte differentiation [166]. Immune activation results in the production of TNF-α and IFN-γ by T cells and TNF-α and IL-6 by monocytes. These pro-inflammatory cytokines were shown to hamper the proliferation of erythrocyte progenitor cells and to antagonize the antiapoptotic activities of EPO. According to studies, the responsiveness of erythrocyte progenitor cells to EPO seems to be inversely correlated with CKD severity as well as the amount of circulating cytokines. EPO requirement to restore the formation of erythrocyte colony-forming units is higher in the presence of elevated levels of IFN-gamma or TNF-α [6]. The inflammatory state contributes to poor response to treatment with EPO which finally leads to cachexia, a higher percentage of patients with cardiovascular disease and reduced quality of life [167,168]. According to studies, the interactions between different inflammatory mediators and ESA responses are complex and it seems that the type of cytokine and its signaling pathway is more important than plasma levels [38]. In the study of hemodialysis patients, epoetin responsiveness was associated with the concentration of IL-6, TNF-α, and IL-12 [59]. Patients in whom levels of TNF-α were ≥2 ng/mL and IL-6 were ≥40 ng/mL required much higher doses of epoetin than patients with lower levels of these cytokines (128 U/kg/week versus 57 U/kg/week; *p* = 0.0024). A negative correlation between IL-12 production and epoetin doses were observed (*p* = 0.029). In turn, Bárány et al. [169] found the relationship between serum C-reactive protein (s-CRP) and the dose of recombinant human EPO required to maintain hemoglobin levels. In their study, weekly EPO dose used in patients with sCRP ≥ 20 mg/L was, on average, 80% higher than in patients with sCRP below that level. Moreover, EPO doses and sCRP inversely correlated with serum albumin and serum iron levels, which imply that the key mechanism through which inflammatory cytokines hamper erythropoiesis is coupled to iron metabolism. A cross-sectional study of maintenance HD outpatients demonstrated a positive correlation between serum concentrations of hs-CRP, IL-6, and TNF-α and both the required epoetin dose and an index of epoetin responsiveness [170]. Large multicenter studies assessing weekly epoetin dose requirement in HD patients categorized into four groups (untreated, hyperresponders, normoresponders, and hyporesponders) on the basis of weekly epoetin dose requirement showed that the median CRP level was higher in the hyporesponders than in the other groups (1.9 versus 0.8 mg/dL; *p* = 0.004) [171]. The median weekly epoetin dose ranged from 30 IU/kg/week in the hyperresponsive group to 263 IU/kg/week in the hyporesponsive group. Ferritin levels were lower in the hyporesponders in comparison to other patients (median 318 versus 445 ng/mL; *p* = 0.01). The results of this analysis support a clear relationship between epoetin hyporesponsiveness and either increased levels of CRP or iron deficiency in HD patients. According to studies, resistance to ESA seems to be related to increased mortality of HD patients, probably due to the fact that are those requiring higher doses of ESA usually have some concomitant infectious, inflammatory, or malignant conditions [170]. Potential strategies targeted at eliminating hyporesponsiveness to ESA in CKD patients with the systemic inflammatory state include selective anticytokine therapy with anti-TNF-α antibodies, IL-1 or IL-6 receptor antagonists, and statins [162,163]. Pentoxifylline, which is a nonselective phosphodiesterase inhibitor exhibiting anti-TNF alpha properties, has been shown to significantly inhibit hemoglobin within six months and reduced serum TNF-α concentration in patients with erythropoietin resistant anemia [172]. Other studies have demonstrated that pentoxifylline decreased other inflammatory parameters, including hsCRP, erythrocyte sedimentation rate (ESR), serum fibrinogen, and TNF-α in patients of CKD [173,174].

## 6. Conclusions

The results of studies have demonstrated that persistent inflammation may contribute to the variability in Hb levels and hyporesponsiveness to ESA which are frequently observed in CKD patients. It seems that variability in Hb values which are often below the target range may contribute to higher morbidity and mortality in these patients [175]. Available evidence implies that chronic kidney disease is a state of the enhanced inflammatory state with high activity of cytokines, which may suppress erythroid progenitor cell production resulting in hyporesponsiveness to ESAs and poor treatment outcomes. The understanding of the impact of inflammatory cytokines on erythropoietin production and hepcidin synthesis will enable to unravel the net of interactions of multiple factors involved in the pathogenesis of the anemia of chronic disease. It seems that anticytokine and antioxidative treatment strategies may be the future of pharmacological interventions aiming at the treatment of inflammation-associated hyporesponsiveness to ESA.

## Figures and Tables

**Table 1:** The summary of trials concerning the use of new drugs in the treatment of anemia in CKD patients.

Study Name	Study Type	Drug Name	Most Important Findings	Ref
DIALOGUE 1 (D1) (*n* = 121)	3 phase 2b, 16-week, randomized, double-blind, placebo-controlled, fixed-dose trial (25, 50, and 75 mg once daily; 25 and 50 mg twice daily) study of molidustat for the treatment of anemia in patients with CKD not previously treated with an analog of rhEPO, and who were not receiving dialysis treatment	Molidustat	Molidustat treatment was associated with estimated increases in mean hemoglobin levels of 1.4–2.0 g/dl	[120]
DIALOGUE 2 (*n* = 124)	Open-label, variable-dose trials, in which treatment was switched from darbepoetin to molidustat or continued with the original agents. Starting molidustat doses ranged between 25–75 mg daily	Molidustat	Hemoglobin levels were maintained within the target range after switching to molidustat, with an estimated difference in mean change in hemoglobin levels between molidustat and darbepoetin treatments of up to 0.6 g/dL.	[120]
DIALOGUE 4 (*n* = 199)	Open-label, variable-dose trials, in which treatment was switched from epoetin to molidustat or continued with the original agents. Starting molidustat ranged between 25–150 mg daily	Molidustat	Hemoglobin levels were maintained within the target range after switching to molidustat 75 and 150 mg, with estimated differences in mean change between molidustat and epoetin treatment of −0.1 and 0.4 g/dL. Molidustat was generally well tolerated, and most adverse events were mild or moderate in severity.	[120]
(*n* = 116)	Randomized placebo-controlled dose-ranging and pharmacodynamics study of roxadustat (FG-4592) to treat anemia in nondialysis-dependent chronic kidney disease (NDD-CKD) patients	Roxadustat	In roxadustat-treated subjects, Hb levels increased from baseline in a dose-related manner. Maximum ΔHb within the first 6 weeks was significantly higher in the 1.5 and 2.0 mg/kg groups than in the placebo subjects. Hb responder rates were dose dependent and ranged from 30% in the 0.7 mg/kg BIW group to 100% in the 2.0 mg/kg BIW and TIW groups versus 13% in placebo.	[114]
(*n* = 143)	Randomized, cohort study with varying roxadustat starting doses and frequencies followed by hemoglobin maintenance with roxadustat one to three times weekly. Treatment duration was 16 or 24 weeks.	Roxadustat	92% of patients achieved hemoglobin response. Higher compared with lower starting doses led to earlier achievement of hemoglobin response. Roxadustat-induced Hb increases were independent of baseline C-reactive protein levels and iron repletion status. Over the first 16 treatment weeks, hepcidin levels decreased by 16.9% (*p* = 0.004), reticulocyte hemoglobin content was maintained, and hemoglobin increased by a mean (±SD) of 1.83 (±0.09) g/dl (*p* < 0.001).	[116]
(*n* = 60)	Open-label, phase IIb study of ESA-naïve incident PD and HD participants (total *n* = 60) with severe anemia (mean Hb 8.3 g/dl at baseline) who were randomized to receive no iron, oral iron, or IV iron during the treatment with roxadustat for 12 weeks	Roxadustat	Roxadustat at titrated doses increased mean Hb by ≥2.0 g/dL within 7 weeks regardless of baseline iron repletion status, C-reactive protein level, iron regimen, or dialysis modality. In groups receiving oral or IV iron, ΔHb(max) was similar and larger than in the no-iron group. Hb response (increase in Hb of ≥1.0 g/dL from baseline) was achieved in 96% of efficacy-evaluable patients. Mean serum hepcidin decreased significantly 4 weeks into study: by 80% in HD patients receiving no iron (*n* = 22), 52% in HD and PD patients receiving oral iron (*n* = 21), and 41% in HD patients receiving IV iron (*n* = 9).	[115]
(*n* = 154)	Phase 3 trial, CKD patients randomly assigned to receive roxadustat or placebo three times a week for 8 weeks in a double-blind manner. The randomized phase of the trial was followed by an 18-week open-label period in which all the patients received roxadustat.	Roxadustat	Hemoglobin level increased by 1.9 ± 1.2 g/dL in the roxadustat group and decreased by 0.4 ± 0.8 g/dl in the placebo group (*p* < 0.001). The mean reduction from baseline in the hepcidin level was 56.14 ± 63.40 ng/mL in the roxadustat group and 15.10 ± 48.06 ng/mL in the placebo group. Hyperkalemia and metabolic acidosis occurred more frequently in the roxadustat group than in the placebo group.	[113]
(*n* = 93)	Phase 2a, multicenter, randomized, double-blind, placebo-controlled, dose-ranging trial (NCT01381094) of adults with anemia secondary to CKD stage 3 or 4. Patients were randomized to 5 groups: 240, 370, 500, or 630 mg of once-daily oral vadadustat or placebo for 6 weeks. All of them received low-dose supplemental oral iron (50 mg daily).	Vadadustat	Vadadustat significantly increased Hb after 6 weeks in a dose-dependent manner in comparison to placebo (*p* < 0.0001). It also increased total iron-binding capacity and reduced ferritin and hepcidin levels.	[132]
	20-week, double-blind, randomized, placebo-controlled, phase 2b study of efficacy and safety of once-daily vadadustat in patients with stages 3a to 5 non-dialysis-dependent CKD	Vadadustat	54.9% of patients on vadadustat and 10.3% of patients on placebo achieved or maintained either a mean hemoglobin level of 11.0 g/dL or more or a mean increase in hemoglobin of 1.2 g/dL or more. Significant rise in reticulocytes and total iron-binding capacity and significant drop in serum hepcidin and ferritin levels were observed in patients on vadadustat compared with placebo. The incidence of adverse events was comparable between the 2 groups.	[129]
(non-dialysis *n* = 71; HD *n* = 80)	Two phase 2a studies to explore the relationship between the dose of daprodustat and hemoglobin response in: - patients with anemia of CKD (baseline hemoglobin 8.5–11.0 g/dL) not undergoing dialysis and not receiving recombinant human erythropoietin (non-dialysis study) - patients with anemia of CKD (baseline hemoglobin 9.5–12.0 g/dL) on hemodialysis and being treated with stable doses of recombinant human erythropoietin (hemodialysis study). Patients were randomized to a once-daily oral dose of daprodustat (0.5 mg, 2 mg, or 5 mg) or placebo for the non-dialysis study; continuing on recombinant human erythropoietin for the hemodialysis study) for 4 weeks, with a 2-week follow-up	Daprodustat	In the non-dialysis study, daprodustat influenced hemoglobin in a dose-dependent (administration of the highest dose resulted in a mean increase of 1 g/dL at week 4)In the hemodialysis study, treatment with daprodustat mean hemoglobin concentrations were maintained in the 5-mg arm after the switch from recombinant human erythropoietin; in lower-dose arms mean hemoglobin decreased. In both studies, the effects on hemoglobin occurred with elevations in endogenous erythropoietin within the range usually observed in the respective populations and markedly lower than those in the recombinant human erythropoietin control arm in the hemodialysis study, and without clinically significant elevations in plasma vascular endothelial growth factor concentrations.	[130]
